# Dupuytren's Disease: A Novel Minimally Invasive Pull-Through Technique

**DOI:** 10.1055/s-0043-1775882

**Published:** 2024-03-04

**Authors:** Michele Maruccia, Pasquale Tedeschi, Francesco Sisto, Ilaria Converti, Giuseppe Giudice, Rossella Elia

**Affiliations:** 1 Division of Plastic and Reconstructive Surgery, Department of Emergency and Organ Transplantation, University of Bari Aldo Moro, Bari, Italy

**Keywords:** Dupuytren, fasciectomy, pull-through, minimally invasive, recurrence

## Abstract

**Background** Dupuytren's disease decreases quality of life significantly and often requires surgical treatment, nevertheless there is no actual gold standard. The aim of this study was to introduce the use of minimally invasive pull-through technique.

**Methods** From 2016 to 2020, 52 patients suffering from Dupuytren's contracture were treated with the minimally invasive pull-through technique. We evaluated the improvement in range of motion, pain, disability, and quality of life in the long term. Total extension deficit, quick disabilities of the arm, shoulder, and hand (QuickDASH), and EuroQol five dimensions—five levels index were systematically scored before each surgical intervention and reevaluated after 24 months.

**Results** Fourteen patients (26.9%) had already received a previous intervention (percutaneous needle aponeurotomy or collagenase *Clostridium histolyticum* ). The mean preoperative total active extension deficit was 84.0 ± 23.3 degrees (55–130 degrees). Mean follow-up was 36 months. There were no cases of tendon rupture or neurovascular injury. Total active extension deficit at the final follow-up was 3.4 ± 2.3 degrees (0–12 degrees). The mean active range of motion of the MCP and PIP joints were, respectively, 90.5 ± 3.3 degrees (85–96 degrees) and 82.7 ± 2.5 degrees (80–87 degrees). At 24 months after cord excision, a mean 10.7 points improvement in the QuickDASH questionnaire was registered ( *p* < 0.001). Pull-through technique was equally effective both on patients with a primary or a recurrent disease. Eight patients (15.4%) had a recurrence of disease in the metacarpophalangeal joint or proximal interphalangeal joint.

**Conclusion** The pull-through technique is a simple, accessible, and effective technique for the treatment of Dupuytren's contracture. The use of palmar mini-incisions combined with minimal dissection has a low risk of iatrogenic injury to the neurovascular bundles and tendons, and has a low risk of recurrence rate. This study reflects level of evidence IV.

## Introduction

Dupuytren's disease is a benign fibroproliferative disorder of the palmar fascia that can significantly impair hand function. 1

Several procedures have been described for the treatment of this pathology, including radical fasciectomy (RF), limited fasciectomy (LF), percutaneous needle aponeurotomy (PNA), and enzymatic fasciotomy through collagenase injections (collagenase *Clostridium histolyticum* [CCH]) with the LF and the PNA as the surgical techniques most commonly performed. Nevertheless, the gold standard treatment is yet to be defined. 2 3

Needle aponeurotomy has recently gained popularity as a minimally invasive technique that can be performed on an outpatient basis. However, this technique is associated with a 5-year recurrence rate of over 80% compared with only 20% with open surgery. 4

Enzymatic fasciotomy with CCH (Xiapex, Swedish Orphan Biovitrum AB, Stockholm, Sweden), although proven to be an effective procedure, has high recurrence rates and complications. 5 Furthermore, in 2019, the drug approved by the European Medicines Agency (EMA) was withdrawn from the European market.

In this scenario, the hand surgeon plays a crucial role having the responsibility to combine digital extension improvement, soft tissue preservation, and disease recurrence prevention in the chosen surgical technique.

The aim of this study was to present our approach for the surgical treatment of Dupuytren's disease through a minimally invasive pull-through technique.

## Methods

A retrospective review of prospectively collected data was performed from January 2016 to January 2020. All patients with Dupuytren's contracture who met the inclusion criteria and underwent minimally invasive pull-through procedure were included in this study.

Inclusion criteria were as follows:

- passive extension deficit > 30 degrees at the metacarpophalangeal joint (MCPJ),
- passive extension deficit > 30 degrees at the MCPJ and more than 20 degrees at the proximal interphalangeal joints (PIPJs),
- stage 1–3 Dupuytren's disease according to Tubiana's classification, 6
- existence of a clearly defined palmar cord,
- recurrence of disease after enzymatic fasciotomy or PNA, and
- patients followed up for at least 24 months.

All patients with isolated PIPJ contracture, stage 4 disease, complications after a previous treatment (infection, neurovascular injury, complex regional pain syndrome), severe osteoarthritis involving MCPJ or PIPJ in the affected finger were excluded from the study.

The Dupuytren's disease was diagnosed by clinical examination, evaluating patient's history and physical tests such as the Hueston's table-top test. 7 A preoperative ultrasound imaging was performed for each patient to evaluate the presence and extent of the pathological tissue.

Patient age, body mass index (BMI), smoking habits, alcohol consumption, diabetes, type of work, family history, the number of fingers involved, joints involved, extent of deformity before treatment, and previous interventions were recorded. Range of movement measurements for each joint were made in the clinic by an experienced hand therapist using a goniometer. These were then used to calculate the extension deficit, which was measured from 0 degrees.

All patients were rigorously followed up with periodic visits at 1, 6, 12, and 24 months.

During the scheduled appointments, a hand surgeon and a hand therapist, not involved in surgical treatment, recorded all reported or observed adverse events. At the 1-month follow-up visit, a sensory evaluation was performed using the two-point discrimination test to assess sensibility of the digits.

The work has been reported to be in-line with Strengthening the Reporting of Observational Studies in Epidemiology guidelines. No donor or funder had a role in the design or conduct of the study, the collection or analyses of the data, or the preparation of the article.

The research complies with the provisions of the Declaration of Helsinki (revised in 2013). The institutional review board of the hospital involved approved this study (No. 6911). Written informed consent was obtained from all patients prior to their enrollment.

### Operative Technique

All operations were performed on an outpatient basis under local anesthesia.

**Fig. 1:**
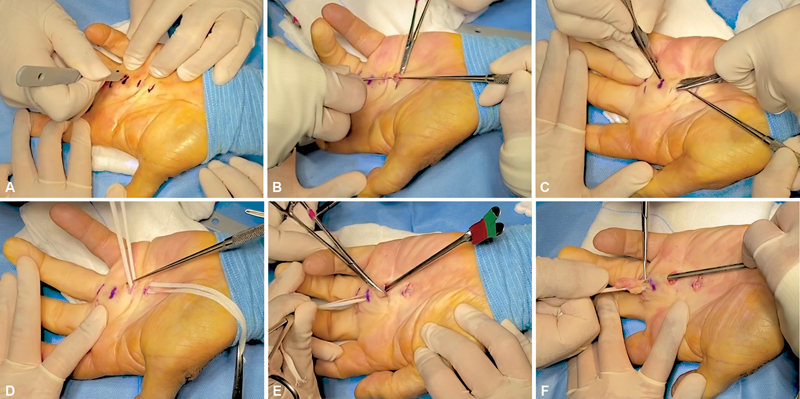
( **A** ) Opening along the preoperative marking of four transverse incisions with a length of 1 cm. ( **B** ) Dissection of the pathological cord from the underlying soft tissues. ( **C** ) Preparation of subcutaneous tunnels between the incisions. ( **D** ) Isolation of the cord from the deep planes with umbilical ribbons. ( **E** ) Protection of the underlying tissues with grooved directors. ( **F** ) Lifting and pulling through the subcutaneous tunnels of the pathological tissue.

After having identified the palpable cords, three to four transverse incisions with a length of 1 cm and spaced approximately 2 to 3 cm from each other were made on the skin overlying the contracture. The incisions were made at the beginning and at the end of the palpable cord, near the distal palmar flexion crease and the digital creases ( Fig. 1A ).

The cords were then isolated from the overlying skin and from the underlying flexor tendons preserving the common and proper palmar digital nerves ( Fig. 1B ). Subcutaneous tunnels were set up between the incisions using blunt-tipped iris scissors ( Fig. 1C ).

The pathological tissue was then isolated from the deep planes with umbilical ribbons ( Fig. 1D ). Grooved directors were inserted through the incisions to protect the vessels of the superficial palmar arch before performing the proximal incision to the retracting cord ( Fig. 1E ). The cords were then lifted and pulled through the previously created subcutaneous tunnels and finally excised ( Fig. 1F ).

In this way, selective fasciotomies were performed aimed at removing the retracting tissue.

The surgical incisions were sutured with absorbable monofilament sutures (monocryl 4/0). If excessive tension was detected at the time of closure, healing by secondary intention was opted.

All patients were provided with a thermoplastic extension splint for 1 week. After 7 days, an overnight splint was adopted to be used for the following month. The splint was molded again to a correct fit at 2 to 3 weeks as local swelling settled.

All patients underwent physiotherapy supervised by a hand therapist.

### Outcome Analysis

The primary endpoint of the study was to evaluate efficacy and durability of the presented technique at 24 months of follow-up. The outcome was defined by the assessment of the correction of the deformity, physical function and symptoms of patients, and the changes in quality of life. Complete correction of the deformity was defined as residual deformity of <5 degrees. 8 Physical function and symptoms of patients were assessed through the quick disabilities of the arm, shoulder, and hand (QuickDASH) system questionnaire. 9 10 Health-related quality of life was evaluated with the EuroQol five dimensions—five levels (EQ-5D-5L) index. 11 A comparison of pre- and postoperative findings was performed. Moreover, a correlation between QuickDASH score and duration of symptoms was investigated.

As additional data, recurrence rate at the latest follow-up was investigated as well as an existent correlation between the preoperative finding (relapsing Dupuytren's disease or primary disease and duration of symptoms) and the outcomes were assessed. Recurrence was defined as the occurrence of >20 degrees of deformity after treatment. 12 13 Moreover, an ultrasound evaluation was performed 12 months after treatment to assess the recurrence of the disease. 8

Surgical complications were recorded. They were divided into early complications, including edema, pain, skin tears, hemorrhage and late complications (wound dehiscence, infection, residual deformity, presence of hypoesthesia, tendon rupture, and complex regional pain syndrome).

### Statistical Analysis

Data were analyzed using the Statistical Package for Social Sciences (SPSS) software for Windows, version 23.0 (IBM SPSS, IBM Corp., Armonk, NY). Confidence interval was set at 95%. Kolmogorov–Smirnov normality test was adopted to assume the normal distribution of statistical variables. Statistical means were compared using the parametric Student's *t* -test (when the normal distribution of variables was assumed) and the nonparametric Wilcoxon's signed-rank test (when the normal distribution of variables was not assumed). Statistical frequencies were compared by means of chi-square test. The strength of correlation between different variables was quantified using Pearson's *r* correlation coefficient. A *p* -value <0.05 was considered statistically significant.

## Results

A total of 52 consecutive patients (40 males and 12 females) with 61 fingers underwent selective fasciectomies with minimally invasive pull-through technique.

Eight patients had concurrent fingers treated in the same sitting. Forty-one fingers had isolated MCPJ involvement while 20 had MCPJ and PIPJ involvement.

Mean follow-up was 36 months from the initial procedure (range: 24–60 months).

The average patient age was 57.5 years (range: 39–78 years) and the mean BMI was 29.7 kg/m ^2^ (range: 26.1–32.3 kg/m ^2^ ). The average duration of symptomatology before the surgical intervention was 14 months. Details regarding patients' characteristics are provided in Table 1 .

**Table 1:** Demographics and patient characteristics

	*n*	Percentage (%)
Number of patients	52	–
Number of fingers	61	–
Age, mean ± SD (range), y	57.5 ± 12.2 (39–78)	
Male	40	76,9
Female	12	23,1
BMI, mean ± SD (range), kg/m ^2^	29.7 ± 2.0 (26.1–32.3)	
Type of worker		
Office worker	13	25.0
Heavy worker	31	59.6
Housewife	8	15.4
Diabetes	11	21.2
Alcohol use	14	26.9
Active smoking	24	46.2
Family history	22	42.3
Hand with contracture		
Right	35	67.3
Left	17	32.7
Number of digits affected, percentage (%)		
1	45	86.5
2	5	9.6
3	2	3.8
Finger involved		
Thumb	0	0
Index	5	8.2
Middle	11	18.0
Ring	19	31.1
Little	26	42.6
Joints involved		
MCPJ	41	67.2
MCPJ and PIPJ	20	32.8
Duration of symptoms ± SD (range), months	14 ± 4.9 (6–24)	
Previous interventions	14	26.9
CCH	8	15.3
PNA	6	11.5
Time to disease recurrence ± SD (range), years	2 ± 0.4 (1.5–2.5)	
Healing process		
First intention	55	90.2
Second intention	6	9.8
Skin graft	0	–

Abbreviations: BMI, body mass index; CCH, collagenase *Clostridium histolyticum* ; MCPJ, metacarpophalangeal joint; PIPJ proximal interphalangeal joint; PNA, percutaneous needle aponeurotomy; SD, standard deviation.

Of the patients included in this study, eight patients had previously undergone treatment with enzymatic fasciotomy, six had undergone PNA, having a disease relapse after approximately 2 years ( Table 1 ).

**Fig. 2:**
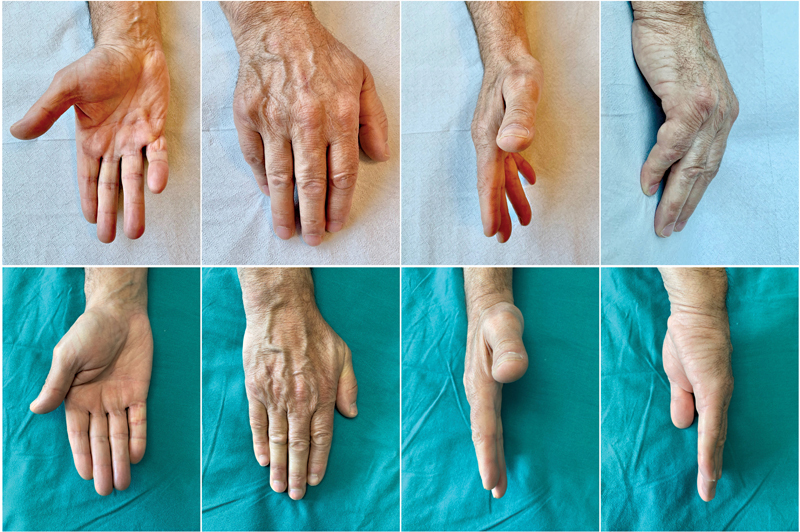
Sixty-one-year-old male with recurrence of Dupuytren's contracture after injection of collagenase *Clostridium histolyticum* . (Above) Preoperative images showing extension deficits in the metacarpophalangeal and proximal interphalangeal joints of the IV and V fingers. (Below) Patient follow-up at 36 months.

The average initial total extension deficit was 47.5 degrees for PIPJ (range: 29–67 degrees) and 53 degrees for MCPJ (range: 32–75 degrees) which were corrected to an average 3.4 degrees (range: 0–12 degrees) after treatment ( Fig. 2 ).

At the last follow-up, the mean active range of motion of the MCP and PIP joints were, respectively, 91.8 ± 2.9 degrees (range: 96–85 degrees) and 83.2 ± 2.1 degrees (range: 87–80 degrees).

Of treated fingers, 86.9% had full correction of the deformity at the metacarpophalangeal and proximal interphalangeal joint. Of the residual deformity, 9.8% was in PIPJ and 3.3% in MCPJ.

Minor wound healing complications were found in 8.2% patients, which improved with regular dressings, and 11.5% of patients experienced pain and swelling after treatment which settled within a month. No patient had tendon tears or neurovascular bundle injuries.

The wound healing process took place in 55 cases by first intention and in six digits by second intention. It was never necessary to resort to a skin graft ( Table 2 ).

**Table 2:** Surgical complications

	Number	Percentage (%)
Early		
Edema	7	11.5
Pain	7	11.5
Skin tear	0	0
Hemorrhage	0	0
Numbness	0	0
Late		
Wound dehiscence	5	8.2
Infection	0	0
Hypo/Anesthesia	0	0
Tendon rupture	0	0
Complex regional pain syndrome	0	0
Residual deformity		
MCPJ	2.0	3.3
PIPJ	6.0	9.8

Abbreviations: MCPJ, metacarpophalangeal joint; PIPJ, proximal interphalangeal joint.

Clinical or instrumental disease recurrence (defined as reappearance of pathological fibromatous tissue in the treated areas) was observed in 15.4% of patients at the latest follow-up. 14

All patients were able to perform the Hueston table-top test at 24 months and returned to their precontracture activities with a mean of 6.2 ± 1.1 (range: 5–8) weeks after surgery. ( Table 3 ). None of the patients exhibited any difficulty in performing flexion–extension movements of the involved digits.

**Table 3:** Patient outcomes and comparison of the pre- and postintervention variables

	*N*	Percentage (%)	*p* -Value
Reduction of MCPJ or PIPJ contracture <5 degrees	53.0	86.9	–
Preoperative total active extension deficit ± SD (range), degrees	84.0 ± 23.3 (55–130)		<0.001
Postoperative total active extension deficit ± SD (range), degrees	3.4 ± 2.3 (0–12)		
Mean active range of motion of the MCP ± SD (range), degrees	90.5 ± 3.3 (85–96)		
Mean active range of motion of the PIP ± SD (range), degrees	82.7 ± 2.5 (80–87)		
Preoperative QuickDASH ± SD (range)			<0.001
Disability/symptoms score	22.8 ± 10.2 (9.1–47.7)		
Postoperative QuickDASH ± SD (range)			
Disability/symptoms score	12.1 ± 6.7 (0–27.3)		
Preoperative EQ-5D-5L index ± SD	0.684 ± 0.3		0.01
Postoperative EQ-5D-5L index ± SD	0.882 ± 0.2		
Recurrence	8	15.4	
Returned to precontracture activities ± SD (range), wk	6.2 ± 1.1 (5–8)		

Abbreviations: EQ-5D-5L index, EuroQol five dimensions—five levels index; MCPJ, metacarpophalangeal joint; PIPJ, proximal interphalangeal joint; QuickDASH, quick disabilities of the arm, shoulder, and hand; SD, standard deviation.

The QuickDASH score improved significantly ( *p* < 0.001) decreasing from a mean value of 22.8 ± 10.2 (range: 9.1–47.7) to 12.1 ± 6.7 (range: 0–27.3) after treatment.

**Fig. 3:**
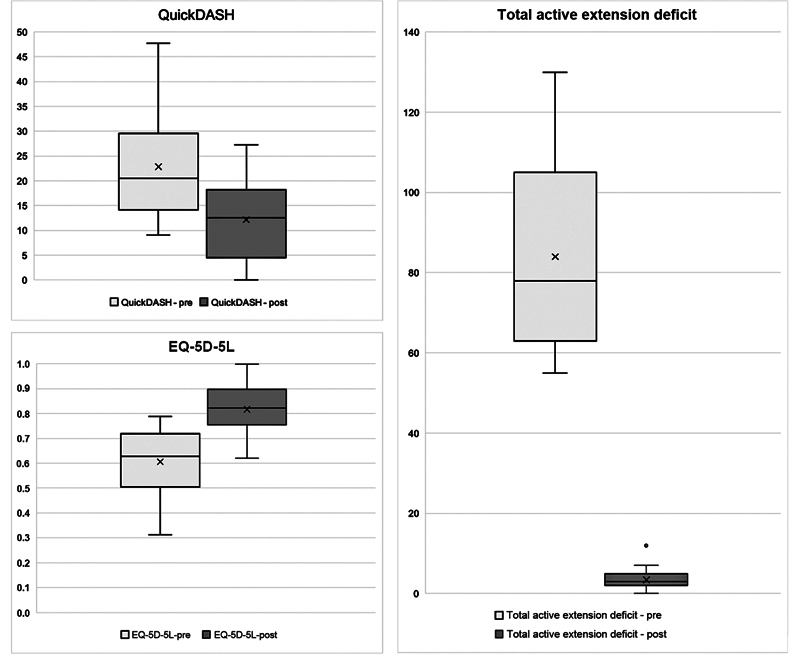
Boxplot of QuickDASH, EQ-5D-5L, and total active extension deficit measurements before and after pull-through technique. All variables improved significantly after treatment. EQ-5D-5L, EuroQol five dimensions—five levels; QuickDASH, quick disabilities of the arm, shoulder, and hand.

Total active extension deficit, and EQ-5D-5L improvement was registered in all patients, *p* < 0.001, *p* = 0.01, respectively. Details and graphics concerning the main outcomes are described in Table 3 and Fig. 3 .

Patients with a relapsing Dupuytren's contracture did not have a difference in improvement compared with patients with a primary contracture.

No correlation was noted between QuickDASH scores and duration of symptoms at the Pearson's *r* collection test. There was a statistically significant correlation between the QuickDASH score and the total active extension deficit ( *r* = 0.652; *p* < 0.001).

## Discussion

Dupuytren's disease is a common problem in most hand surgery practices. It is usually easily diagnosed by the presence of its primary palmar manifestations: the nodule, the cord, and the digital flexion contracture.

In 1991, Smith described what the three goals of treatment for Dupuytren's contracture were, precisely, to improve the functional capacity of the affected hand, reduce the deformity associated with contractures, and ultimately prevent disease recurrence. 15

Nowadays, we would emphasize another goal of treatment: the preservation of noble structures like the common and proper palmar digital nerves, the flexor tendons, and digital arteries.

Duputyren's disease poses a real challenge for many hand surgeons since no standard consensus exists on the procedure to be performed. Several factors must be considered in the treatment decision, including the severity of the disease, the risk of complications, and the rate of relapse. 16

RF and dermatofasciectomy have fallen out of favor as more aggressive techniques, with higher complication rates, often requiring skin grafts and a prolonged postoperative rehabilitation process. 17

Percutaneous needle aponeurotomy (PNA) is a technique that has gained popularity in the recent period, as it is a simple procedure, which can be performed under local anesthesia on an outpatient basis. The main complications of PNA are temporary digital numbness and tendon tears. Additionally, there may be a greater risk of disease recurrence, as the weakened pathological tissue remains present at the site. 18 19 Van Rijssen et al compared LF and percutaneous fasciotomy in 5-year randomized clinical trial, demonstrating that PNA has a recurrence rate of 84.9%, compared with 20.9% for fasciectomy. 20 In a separate study, Van Rijssen et al reported that passive extension deficit was significantly improved in those with advanced Tubiana staging who underwent fasciectomy. 21 PNA allows for good recovery of hand function, but full extension (<5 degrees) is usually not achieved. 22

Another minimally invasive alternative approach to Dupuytren's contracture was the use of the CCH (Xiapex, Swedish Orphan Biovitrum AB). It produced comparable results to LF with improved effectiveness when administered to the MCPJ compared with PIPJ contractures. 23 24 However, the EMA withdrew Xiapex from the European market in 2019. 25 Hurst showed that recurrence with recurrent contractures more than 20 degrees was 35% at 3 years and 47% at 5 years. 26 It was an expensive treatment and had complications related to collagenase injection including localized swelling, pain and bruising, itching, tenderness, skin tears, and transient lymphadenopathy, and less commonly complex regional pain syndrome and tendon rupture. 27 28

To the best of our knowledge, a minimally invasive pull-through technique has not been previously described as a minimally invasive surgical treatment for Dupuytren's disease. In our analysis, the described technique demonstrated to be effective resulting in a complete correction in 86.9% of patients. The difference in finger range of motion between pre- and postoperative was shown to be statistically significant ( *p* < 0.05). Furthermore, the comparison of the data collected through the QuickDASH and EuroQol 5-dimension questionnaires before and after treatment confirmed the validity of the recovery of hand function after treatment ( *p* < 0.05). Improvements registered after pull-through technique were neither correlated to the symptoms' duration, nor were the differences in patients who had already undergone a previous treatment. Compared with the enzymatic treatment, our approach, although more complex and invasive, reported a low recurrence rate and none of the complications associated with the collagenase. 29 30 31 Conversely, no major adverse events were reported in our study population.

Recurrence rate was 15.4%, indicating that excising the diseased fascia of affected digits and palm is very effective for treating contractures with a relatively low long-term recurrence rate. The hypothesis is that reducing the extent of incisions and dissection would also reduce the production of collagen that can cause further retraction and disease recurrence.

Considering our results as a whole, we believe that the minimally invasive pull-through technique has several advantages. First, it is an open technique, therefore it allows to visualize and to preserve the anatomical structures that need to be protected, such as the common and proper palmar digital nerve and the flexor tendon. Second, the role of the mini-incisions is crucial, as they allow the surgeon to extract the cord without the need for a larger and more elaborate dissection such as that required in LF with Bruner incisions decreasing operative time.

Setting up subcutaneous tunnels during the procedure requires meticulous attention to avoid any damage to the skin. In our experience, we have found that it is possible to separate the skin from the fibrotic tissue without encountering real ulcerations. We acknowledge that the risk of thin skin flaps is present, but we have not observed any skin necrosis in our patient cohort. We believe that the absence of stitches in cases of extreme tension reduces the ischemia of the margins and helps to minimize the risk of complications.

The 12-month ultrasound evaluation allowed us to perform a comprehensive assessment of tendon gliding following the operative technique. This enabled us to observe any residual deformities that may have remained following treatment. Overall, the inclusion of ultrasound evaluation in our study allowed for a more thorough evaluation of the effectiveness of the procedure.

Other minimally invasive surgical techniques have been described in the literature, however, based on segmental aponeurectomies and not on “pull-thorugh” complete fasciectomies. 32 33 34

Attention should be drawn to the anatomical differences that the pathology could have at the PIP joint. At this level it is possible to encounter retrovascular or spiral cords. The neurovascular bundle often courses between contracted Grayson's ligaments and the lateral cords. These conditions can be problematic as digital neurovascular bundles are often displaced central, proximal, and superficial. In these tough cases, if it is not possible to easily separate the retrovascular cord from the neurovascular bundle, the surgical access could be widened joining the two transverse incisions at the level of the PIPJ, making a modified Bruner-type incision. In this way, the surgeon can always rely on a backup plan in case of troubles, switching from minimally invasive technique to a full-open approach with adequate visualization of the neurovascular bundle. All patients in which the incision was widened into a Z fashion were excluded by this study. Proper patient selection and careful consideration of potential risks and complications are important for a successful outcome.

Based on our results, this novel minimally invasive approach is a safe and effective alternative treatment for Dupuytren's contracture, allows the surgeon to deal with cases of advanced contractures as well as relapses, and could represent an added value for the functional outcome of patients.

The limitations of the study are to be found in the retrospective design, in the heterogeneity of the study population, lack of a control group, and in the need for a longer follow-up. This technique requires further validation with a multicenter study and comparative trial with other procedures.
